# The Short-Term Effects of Rhythmic Vibrotactile and Auditory Biofeedback on the Gait of Individuals After Weight-Induced Asymmetry

**DOI:** 10.33137/cpoj.v5i1.36223

**Published:** 2022-02-07

**Authors:** A. Michelini, H. Sivasambu, J. Andrysek

**Affiliations:** 1 Institute of Biomedical Engineering, University of Toronto, Toronto, Canada.; 2 Bloorview Research Institute, Holland Bloorview Kids Rehabilitation Hospital, Toronto, Canada.

**Keywords:** Spatiotemporal Gait Asymmetry, Biofeedback, Rehabilitation, Entrainment, Amputation, Rhythmic Stimulation, Gait

## Abstract

**BACKGROUND:**

Biofeedback (BFB), the practice of providing real-time sensory feedback has been shown to improve gait rehabilitation outcomes. BFB training through rhythmic stimulation has the potential to improve spatiotemporal gait asymmetries while minimizing cognitive load by encouraging a synchronization between the user’s gait cycle and an external rhythm.

**OBJECTIVE:**

The purpose of this work was to evaluate if rhythmic stimulation can improve the stance time symmetry ratio (STSR) and to compare vibrotactile to auditory stimulation. Gait parameters including velocity, cadence, stride length, double support time, and step length symmetry, were also examined.

**METHODOLOGY:**

An experimental rhythmic stimulation system was developed, and twelve healthy adults (5 males), age 28.42 ± 10.93 years, were recruited to participate in walking trials. A unilateral ankle weight was used to induce a gait asymmetry to simulate asymmetry as commonly exhibited by individuals with lower limb amputation and other clinical disorders. Four conditions were evaluated: 1) No ankle weight baseline, 2) ankle weight without rhythmic stimulation, 3) ankle weight + rhythmic vibrotactile stimulation (RVS) using alternating motors and 4) ankle weight + rhythmic auditory stimulation (RAS) using a singletone metronome at the participant’s self-selected cadence.

**FINDINGS:**

As expected the STSR became significantly more asymmetrical with the ankle weight (i.e. induced asymmetry condition). STSR improved significantly with RVS and RAS when compared to the ankle weight without rhythmic stimulation. Cadence also significantly improved with RVS and RAS compared to ankle weight without rhythmic stimulation. With the exception of double support time, the other gait parameters were unchanged from the ankle weight condition. There were no statistically significant differences between RVS and RAS.

**CONCLUSION:**

This study found that rhythmic stimulation can improve the STSR when an asymmetry is induced. Moreover, RVS is at least as effective as auditory stimulation in improving STSR in healthy adults with an induced gait asymmetry. Future work should be extended to populations with mobility impairments and outside of laboratory settings.

## INTRODUCTION

Spatiotemporal gait asymmetry is a condition commonly exhibited in clinical populations with mobility difficulties including individuals with lower limb amputation (LLA), Parkinson’s disease, and cerebral palsy. Gait asymmetries result in atypical biomechanical and walking and loading patterns, and over time, can lead to long-term musculoskeletal issues such as joint degeneration and osteoarthritis.^1,2^ Excessive gait deviations and asymmetry can be attributed to a lack of proper gait training leading to the development of poor gait habits.^1^ However, there are limitations to conventional in-person gait training sessions with a physiotherapist, such as the cost and accessibility of the service.^3^ Modern approaches and technologies such as virtual reality, rehabilitation video games, and biofeedback (BFB) systems take advantage of motor learning strategies and are promising tools for gait rehabilitation in the clinic and at home.^4^

BFB is the practice of providing real-time feedback to an individual based on collected information from that user.^5^ BFB can be used to supplement traditional gait training using various modalities – most commonly through visual, auditory, and vibrotactile. Auditory and vibrotactile feedback modalities are most suited for wearable and field-based applications, however, there is no clear consensus on the most appropriate modality for gait rehabilitation.^5^ Both modalities are commonly utilized as part of sensory augmentation and substitution. Specifically, in the case of gait rehabilitation, auditory or tactile stimulation modalities indirectly provide information about gait movements and events. While both modalities act to augment sensory feedback, they do so by utilizing different neural physiology and pathways.^6,7^ This can manifest into unique responses or levels of biofeedback effectiveness.

Rhythmic movement interventions are a form of BFB that have been shown to improve automaticity and gait regularity.^8^ Entrainment is the phenomenon whereby two out of phase rhythms synchronize.^9^ The practice of using entrainment for gait training has been shown to be successful for a variety of populations, where the individual will synchronize their gait cycle (heel strike or toe-off times) to an external beat or tempo.^10^ This can be accomplished through rhythmic auditory stimulation (RAS). RAS has been shown to improve cadence and symmetry and can be effective for gait recovery.^11^ It has also been shown to reduce stride time, swing time, and step time variabilities for individuals with Parkinson’s disease and following stroke.^12^ Further potential benefits include increased cadence and gait symmetry.^13^

For individuals with LLA, RAS has been shown to decrease gait training times.^14^ Compared to its auditory counterpart, the effects of rhythmic vibrotactile stimulation (RVS) are not as well understood; however, RVS is a promising modality for gait rehabilitation and particularly wearable applications since it does not interfere with the auditory system. RVS has been shown to improve step length, and cadence in patients with Parkinson’s disease using vibration motors at the wrist^13^ and ankle.^14^

The overall goal of this study was to evaluate and compare the efficacy of vibrotactile and auditory rhythmic stimulation to improve the stance time symmetry ratio (STSR) of able-bodied individuals with induced asymmetries. Increased stance time symmetry has been associated with improved gait performance and rehabilitation outcomes in certain patient groups.^15–17^ Secondarily, the study examined other key gait parameters including velocity, cadence, stride length, double support time, and step length symmetry associated with rhythmic stimulation.

## METHODOLOGY

### System Instrumentation

**Figure 1:**
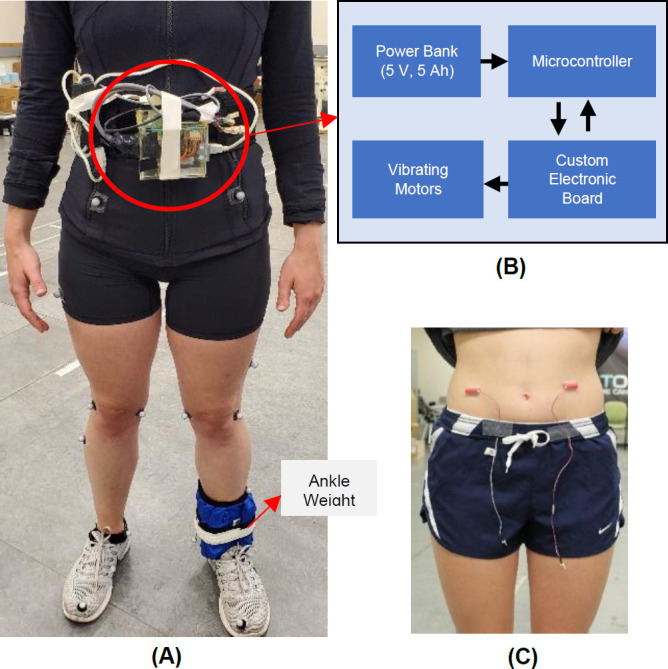
Equipment and Instrumentation for RVS system. **A**: Setup of the BFB system on a participant; **B**: Microcontroller-based Control Unit, including the custom electronic board, and power supply; **C**: Vibrating Unit (motors) located at the lower abdomen.

**Figure 2:**
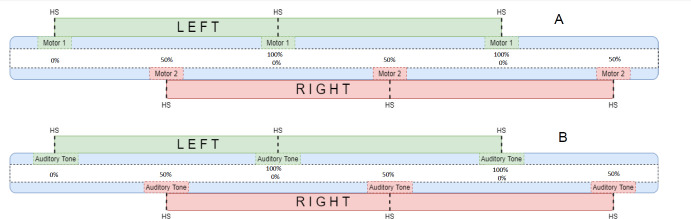
Rhythmic Vibrotactile Stimulation Delivery; **B**) Rhythmic Auditory Stimulation Delivery. Note: HS (Heel Strike) and TO (Toe-Off) indicate when the user should be in the HS and TO portions of the gait cycle, respectively, if following the rhythmic stimulation.

A wearable microcontroller-based system was developed to provide vibrational stimulation at the user’s preferred cadence and target STSR. RVS was delivered using two 9 mm vibration motors (Model 307-103-Precision Microdrives Ltd, London, United Kingdom) for a duration of 100 ms. Each vibrating motor was supplied with 3.3 V, corresponding to a nominal vibration frequency of 250Hz and vibration amplitude of 7.5 G. An Arduino UNO (Arduino, Somerville, Massachusetts) was used to control the timing of the RVS delivery and was placed on the user’s waist as shown in **Figure 1** using a Velcro waist strap. The system was powered by a single cell 5000 mAH Lithium-ion battery. A motor was adhered directly to the skin on each side of the user’s lower abdomen as per Crea et al.,^18^ behind the waist strap. Recent work has found that higher frequency vibrations (>230 Hz), targeting Ruffini cylinders and Pacinian corpuscles skin mechanoreceptors, increase user detection accuracy and reduce reaction times following vibrotactile stimulation.^19,20^ RVS alternated between the left and right sides using the two motors as described in **Figure 2a**.

RAS was delivered using a digital metronome (Google, Mountain View, California) on a PC speaker loud enough such that the participant could hear throughout the gait laboratory. The auditory tones were delivered using a singular tone for both left and right limbs as described in **Figure 2b**.

### Participants

A convenience sample of twelve (N = 12) able-bodied adults were recruited for this study. Inclusion criteria included: 1) above the age of 18 years, 2) English speaking, and 3) having no physical or gait-related impairments, ambulation difficulties, or neuro-motor compromises. The study was approved by the Research Ethics Board at Holland Bloorview Hospital, Canada. Informed written consent was obtained from each participant before commencing.

### Experimental Setup and Data Processing

Previous research has shown that when using rhythmic cueing to improve gait parameters such as stride length and cadence, it is most effective when the provided tempo of the stimulation is close to the participant’s natural cadence.^21,22^ For this reason, the cadence was determined by having the participant walk at a self-selected speed without the ankle weight. The tempo of the RVS and RAS was set to the identified cadence value at the user’s self-selected baseline speed.

To induce asymmetry of gait parameters an ankle weight of 2.27 kg was used; an intermediate value to that used in other studies (1.95 – 3 kg),^23,24^ placed on the non-dominant leg.^25^ The ankle weight was placed on the non-dominant leg to compound the asymmetric effect. To determine leg dominance, the participant was asked which leg they use to kick a soccer ball.^26^ The participant was given five minutes to adjust to the added ankle weight as per Smith and Martin.^23^

Gait data were collected with the Cortex software (Motion Analysis Corporation, Santa Rosa, California) using a system with 12 cameras, sampled at 200 Hz. Twenty retroreflective markers were placed following a modified Helen Hayes Lower Extremity Marker: medial and lateral ankles, toes, heels, tibias, medial and lateral knees, thighs, anterior superior iliac spines, sacral, and right offset.^27,28^ When the ankle weight was added, the lateral and medial ankle markers were placed on the outside of the ankle weight and in line with the unloaded lateral and medial ankle markers. The motion capture position data were smoothed within the Cortex software applying a Butterworth low pass filter with a cut-off frequency of 6 Hz, as per the study by Schreiber and Moissenet.^29^ Gait parameters were processed automatically using the Cortex Software.

### Protocol

Data for each participant were collected during a single twohour session. The participant was asked to walk at a self-selected speed back and forth along an 8 m walkway located in a gait laboratory. A single walking trial consisted of one pass of the 8 m walkway. Five baseline trials were collected. On average, 1-2 full gait cycles were recorded on each pass of this walkway. Following the baseline trials, the researcher analyzed one of the trials to determine the participant’s preferred cadence. The participant was then instrumented with the unilateral ankle weight, given 5 minutes to walk and adjust to the weight, and then provided a 5-minute break to avoid fatigue. Next, rhythmic stimulation was provided. The order of RVS or RAS trials was randomized using simple and balanced randomization through a random number generator (1= RVS first, 2 = RAS first). The participant was instructed to walk to the tempo of the RVS or RAS, with their heel strike occurring at the time of the stimulus. Five minutes of practice with the stimulation was followed by a 5-minute break. Subsequently, 5 walking trials with rhythmic stimulation were collected. This process was repeated with the other stimulation modality.

### Outcome Measures

The primary outcome measure for this experiment was stance time symmetry ratio (STSR), calculated using *Equation 1*, where the limb with the unilateral ankle weight is considered the loaded limb, while the limb without the ankle weight is considered the unloaded limb. STSR was used because of its demonstrated ease of interpretation by the user and the ability to assess gait control through this variable.^30^ Perfect symmetry is equal to a value of 1, while asymmetry is less than or greater than 1. The individual spends more time in the stance portion of the gait cycle on the unloaded limb if the symmetry ratio is less than 1. Some deviation around the value of 1 is considered normal. For example, Patterson et al. found that for healthy participants, the mean STSR was 1.02 ± 0.02 with an upper 95% confidence interval boundary of 1.05.^31^

Secondary outcome measures included cadence, forward velocity, double support time, step length symmetry ratio (calculated using the same formula as STSR), and stride length.


Equation (1) $$STSR=\frac{Stance\;Time_{loaded\;limb}}{Stance\;Time_{unloaded\;limb}}$$

### Statistical Analysis

Statistical analyses were performed using JMP Pro Software (Statistical Discovery, SAS, USA). The data were tested for normality using the Shapiro-Wilk W test for each set (*p*<0.05). Using a repeated measures multivariate analysis of variance (rMANOVA), the outcome measures were compared among baseline, ankle weight without rhythmic stimulation, RVS, and RAS. To account for Type I error, a Bonferroni Correction was applied to the original α value of 0.05. Therefore, the significance level of α = 0.05/6 = 0.0083 was used for all statistical tests. The order of RVS and RAS was applied in the rMANOVA as an effect to account for training throughout the experiment. A paired ttest was applied for post hoc analysis. If p<0.0083, we rejected the null hypothesis that:


$$H_{o}:\mu_{baseline}=\mu_{ankle\;weight\;baseline}=\mu_{RVS}=\mu_{RAS}.$$

Effect sizes were calculated using partial η^2^ with effect sizes of 0.01, 0.06, and >0.14 considered small, medium, and large, respectively. Mauchly’s Test of Sphericity was used to test for the assumption of sphericity.^32^

### RESULTS

The participant characteristics are shown in **Table 1**.

**Table 1:** Participant Characteristics.

Variable	Range	Mean ± StandardDeviation
Age (years)	23–61	28.4 ± 10.9
Height (cm)	167–183	174.6 ± 4.7
Weight (kg)	58.5–113	71.6 ± 15.7
Calculated Cadence (steps/min)	103–118	111.9 ± 4.7
Sex: M/F	M: 5, F: 7	
Ankle Weight Placement: Left/Right	Left: Right: 1, Left: 11	

Mauchly’s Test of Sphericity indicated that the assumption of sphericity had been violated (p<0.05) for cadence [χ^2^(5) = 21.48, p<0.0001], stride length [χ^2^(5) = 14.88, p = 0.011], velocity [χ^2^(5) = 14.66, p = 0.012], and step length ratio [χ^2^(5) = 19.81, p = 0.0.0014], therefore degrees of freedom were corrected using Greenhouse-Geisser estimates of sphericity. The rMANOVA showed significance for STSR [F(3,9) = 55.5, p <0.0001], double support time [F(3,9) = 17.63, p = 0.0004] and cadence [F(1.91, 21.05) = 17.86, p<0.0001], while step length ratio [F(1.43, 15.72) = 0.86, p = 0.41], stride length [F(1.67,18.42) = 0.99, p = 0.38] and velocity [F(1.78,19.56) = 4.45, p = 0.035] were not. The corresponding effect sizes were 0.95, 0.854, 0.731, 0.001, 0.011 and 0.012, respectively. There was no order effect in the experiment. Means and standard deviations for the 4 conditions and 6 parameters are found in **Table 2**. Based on the post hoc tests, there was a significant decrease in STSR from the no ankle weight baseline to ankle weight without rhythmic stimulation conditions [t(11) = −11.99, p<0.0001]. The total STSR decrease was 0.061 ± 0.018. Significant differences were also found between baseline and RVS [t(11) = −11.68, p<0.0001], and baseline and RAS conditions [t(11) = −13.09, p<0.0001]. There was also a significant improvement from ankle weight without rhythmic stimulation to RVS condition [t(11) = 4.91, p = 0.005]. Further, there were significant differences in two of the secondary outcome measures, including cadence, and double support time (**Table 2**).

**Table 2:** Gait Parameters Under Different Conditions.

	No ankle weight baseline	Ankle weight without rhythmic stimulation	Ankle weight with rhythmic vibrotactile stimulation (RVS)	Ankle weight with rhythmic auditory stimulation (RAS)
	(Mean ± Standard Deviation)			
Stance Time Symmetry Ratio	0.999 ± 0.007^† ‡^	0.938 ± 0.019^* ‡^	0.952 ± 0.016^* †^	0.950 ± 0.014^*^
Cadence (steps/minute)	111.9 ± 5.2^†^	107.9 ± 5.5^* ‡^	111.7 ± 4.4^†^	112.5 ± 4.3^† ‡^
Step Length Symmetry Ratio	0.998 ± 0.025	0.994 ± 0.055	1.006 ± 0.023	1.009 ± 0.019
Stride Length (cm)	139.6 ± 11.6	140.8 ± 13.9	142.4 ± 15.9	142.8 ± 15.4
Velocity (cm/s)	130.1 ± 11.5	126.8 ± 14.9	132.7 ± 16.6	133.9 ± 15.6
Double Support Time (% of the gait cycle)	32.7 ± 1.7^† ‡^	31.2 ± 2.1^* ‡^	30.3 ± 2.6^* †^	30.1 ± 2.2^* †^

^*^ Denotes significantly different values than the baseline condition

^†^ Denotes significantly different values than the ankle weight without rhythmic stimulation condition

^‡^ Denotes significantly different values than the RVS condition

Post hoc analysis showed a significant decrease in cadence occurred from baseline to ankle weight without rhythmic stimulation conditions t(11) = −4.37, p = 0.0011. There was also a significant increase in cadence from ankle weight without rhythmic stimulation to RVS conditions t(11) = 5.45, p = 0.0002, ankle weight without rhythmic stimulation to RAS condition t(11) = 5.25, p = 0.0003, and RVS to RAS t(11) = 3.01, p = 0.0117. Significant differences were found between double support times at baseline and ankle weight without rhythmic stimulation condition t(11) = −5.42, p = 0.0002, baseline and RVS condition t(11) = −6.17, p<0.0001, baseline and RAS t(11) = −7.76, p< 0.0001, ankle weight without rhythmic stimulation and RVS t(11) = −3.15, p = 0.0093, and ankle weight without rhythmic stimulation and RAS t(11) = −4.14, p = 0.0016.

## DISCUSSION

This study has uniquely compared auditory and vibrotactile stimulation and shown that RVS may be at least as effective as its auditory counterpart in improving walking symmetry, as well as cadence while maintaining other gait parameters (with the exception of double support time). This presents an important step in the development of wearable biofeedback systems to augment the gait rehabilitation of individuals with mobility impairments.

These findings align with previous studies involving both clinical and healthy populations. In this study the addition of an ankle weight provided a means to simulate asymmetrical gait. Without any form of rhythmic stimulation the weight significantly reduced STSR via an elongation of step time of the loaded limb.^24,25^ Rhythmic stimulation was then provided resulting in improved gait symmetry. A similar effect has also been found in clinical populations, namely stroke patients.^33–35^

Although biofeedback significantly improved symmetry in this study, perfect symmetry was not achieved. The inability to achieve a greater change in STSR and/or perfect symmetry is likely related to limitations of the person’s capabilities; in the case of clinical populations, this may, for example, include motor control issues or limited muscle strength, associated with the disability. Similarly, the healthy individuals in this study were likely not able to fully overcome the effects of the ankle weight. Not only may perfect symmetry be unachievable in some cases, but it may also be undesirable. In the case of someone with a physical or biomechanical asymmetry (i.e. contralateral limbs of different masses such in the case of a lower-limb prosthetic user, or stroke patient with muscle weakness on one side), a slightly asymmetrical gait may present a more optimal walking pattern (i.e. to decrease metabolic cost or increase stability).^36^ Hence, the application of biofeedbackbased gait training must carefully consider the patient’s capabilities as well as rehabilitation goals.

Both gait velocity and gait symmetry are commonly used to measure overall gait performance as indicators for community ambulation and level of gait control, respectively.^31^ Robinson and Smidt note that as gait velocity increases, so does overall mobility.^37^ In our previous research using corrective biofeedback, symmetry was achieved at the cost of decreased walking speed and cadence;^38^ in contrast, this experiment demonstrated greater symmetry (using both biofeedback modalities) accompanied by a significant increase in cadence, while other measured spatiotemporal parameters remained unchanged from the baseline condition. The lack of significant change in velocity, stride length and step length symmetry ratio may be a result of the biofeedback targeting temporal rather than spatial aspects of gait. Hence vibrotactile rhythmic stimulation, like its auditory counterpart, applied in this experiment has the potential to improve gait more holistically.

Both stimulation methods (vibrotactile and auditory) produced similar results, and improvements in gait. This was not necessarily an expected finding, since the sensory systems utilize different receptors, neural pathways and processing centers. Sigrist et al. has said that auditory feedback is suitable for the perception of temporal information, while haptic feedback is appropriate for the perception of spatial and temporal information.^6^ Moreover, in everyday life, humans are exposed to auditory and vibrotactile rhythmic stimuli differently, hence one might expect that responses would differ also. For example, music is a common stimulus resulting in entrainment and the synchronization to auditory signals.^39^ Entrainment based on somatosensory stimulation on the other hand, is less common in the physical world. Hence, one may expect auditory stimuli to be more effectively utilized. This study, however, suggests that the sensory modality may play a minor role, as long as signals are able to be adequately sensed. Further, it may be possible that gait performance is more substantially influenced by elements of motor control or even perhaps biomechanics (ability of our muscles to control the movements to overcome the effects of the ankle-weight) rather than the ability to sense and process rhythmic stimulation. Future work is needed to better elucidate the neurophysiological mechanisms involved in the utilization of biofeedback in gait.

The findings of this experiment may be influenced by several factors. Increased double support times were exhibited across all conditions in this study compared to typical gait of 20%.^40^ This may be due to slower gait or gait compensations that were caused by the test conditions. Modality selection for providing feedback through sensory substitution is a continuously developing field as the underlying mechanisms and resulting effectiveness for specific applications are uncovered.^7^ Work performed in the field of balance control found varying effectiveness and latencies in responding to feedback modalities based on age, proposing that decreased residual processing capacity may affect one’s ability to respond to cues from feedback systems.^7,41^ In case future research confirms auditory and vibrotactile stimulation to produce similar neurophysiological responses, the ultimate decision about which modality to use in biofeedback systems may hinge on other factors such as cost and ease of implementation.

### Study Limitations

This study has several limitations. One limitation of this study is that the sound from the motors was not controlled for in this experiment. One study that used rhythmic haptic stimulation had the participants wear headphones with white noise so that they would avoid entrainment with the sound of the vibrations and external stimuli.^42^ However, self-generated auditory feedback such as footsteps when walking is an important factor when controlling spatial and temporal parameters,^43^ therefore headphones with white noise were not used to mask the sounds from the vibrotactile stimulation in our experiment. Additionally, the mass of the ankle weight used for all participants was equal regardless of body mass index, leg strength or other factors. In future work the ankle weight should be adjusted based on the participant’s characteristics to achieve a similarly difficult experience for all subjects.

### Opportunities for Future Research

Future experiments should also collect and analyze kinematic data, which would have been useful in identifying any further compensatory mechanisms that the individual exhibited. Future work should provide more than one gait training session and assess the retention values and long-term efficacy of RVS and RAS. In terms of prototype development, testing of the RVS system should be conducted outside of controlled settings to better characterize aspects related to cognitive loading and impacts of environmental factors on the user’s ability to effectively use the system.

Although it was important to first test this system on able-bodied adults with an induced asymmetry, future work should evaluate the effectiveness of RVS and RAS on other clinical populations, such as individuals with LLA. With clinical populations, other therapy goals must be addressed before and during gait training such as range of motion, muscle strength, stability, and proprioception. Further, studies are needed to inform the patient demographics that may benefit from the biofeedback system. Not all patients may be able to utilize or appropriately respond to biofeedback, and in some cases targeting gait improvements may not align with or be a rehabilitation goal.

## CONCLUSION

This study has shown that both rhythmic vibrational stimulation and rhythmic auditory stimulation can effectively improve walking asymmetry induced by the addition of an ankle weight. In addition to improving stance time symmetry ratio, other important aspects of gait such as cadence were preserved. Establishing the viability of vibrotactile based gait training and BFB systems is an important step in developing technologies and feedback modalities for use outside of clinical settings where auditory feedback may not be appropriate.

## DECLARATION OF CONFLICTING INTERESTS

Contents from this manuscript were part of a master’s thesis. The authors declared no potential conflicts of interest with respect to the research, authorship, and/or publication of this article.

## AUTHOR CONTRIBUTION

**Alexandria Michelini:** Conceptualization, methodology, validation, formal analysis, investigation, writing original draft, writing/review and editing, visualization.

**Harry Sivasambu:** Validation, formal analysis, investigation, writing/review and editing, visualization.

**Jan Andrysek:** Supervision, conceptualization, methodology, investigation, writing/review and editing.

## SOURCES OF SUPPORT

The project was supported by Ontario Graduate Scholarship and by NSERC CRD (CRDPJ 491125).

## ETHICAL APPROVAL

The study was approved by the Research Ethics Board at Holland Bloorview Hospital, Canada. Informed written consent was obtained from each participant before commencing.

## References

[1] Gailey R. Review of secondary physical conditions associated with lower-limb amputation and long-term prosthesis use. J Rehabil Res Dev. 2008; 45:15–30. DOI: 10.1682/JRRD.2006.11.0147 18566923

[2] Nolan L, Wit A, Dudziñski K, Lees A, Lake M, Wychowañski M. Adjustments in gait symmetry with walking speed in trans-femoral and trans-tibial amputees. Gait Posture. 2003; 17:142–51. DOI: 10.1016/S0966-6362(02)00066-8 12633775

[3] Parker J, Mountain G, Hammerton J. A review of the evidence underpinning the use of visual and auditory feedback for computer technology in post-stroke upper-limb rehabilitation. Disabil Rehabil Assist Technol. 2011; 6:465–72. DOI: 10.3109/17483107.2011.556209 21314295

[4] Escamilla-Nunez R, Michelini A, Andrysek J. Biofeedback systems for gait rehabilitation of individuals with lower-limb amputation: A systematic review. Sensors. 2020; 20:1628. DOI:10.3390/s20061628 32183338 PMC7146745

[5] Van Gelder LMA, Barnes A, Wheat JS, Heller BW. The use of biofeedback for gait retraining: A mapping review. Clin Biomech. 2018; 59:159–66. DOI: 10.1016/j.clinbiomech.2018.09.020 30253260

[6] Sigrist R, Rauter G, Riener R, Wolf P. Augmented visual, auditory, haptic, and multimodal feedback in motor learning: A review. Psychon Bull Rev. 2013; 20:21–53. DOI: 10.3758/s13423-012-0333-8 23132605

[7] Sienko KH, Seidler RD, Carender WJ, Goodworth AD, Whitney SL, Peterka RJ. Potential mechanisms of sensory augmentation systems on human balance control. Front Neurol. 2018; 9. DOI:10.3389/fneur.2018.00944 PMC6240674 30483209

[8] Bridenbaugh SA, Kressig RW. Laboratory Review: The role of gait analysis in seniors’ mobility and fall prevention. Gerontology. 2011; 57:256–64. DOI: 10.1159/000322194 20980732

[9] Moumdjian L, Buhmann J, Willems I, Feys P, Leman M. Entrainment and synchronization to auditory stimuli during walking in healthy and neurological populations: A methodological systematic review. Front Hum Neurosci. 2018; 12. DOI:10.3389/fnhum.2018.00263 PMC6028729 29997491

[10] Ghai S, Ghai I, Effenberg AO. Effect of rhythmic auditory cueing on aging gait: a systematic review and meta-analysis. Aging Dis. 2018; 9:901. DOI:10.14336/AD.2017.1031 30271666 PMC6147584

[11] Chamorro-Moriana G, Moreno A, Sevillano J. Technologybased feedback and its efficacy in improving gait parameters in patients with abnormal gait: A systematic review. Sensors. 2018; 18:142. DOI:10.3390/s18010142 29316645 PMC5795813

[12] Wright RL, Bevins JW, Pratt D, Sackley CM, Wing AM. Metronome cueing of walking reduces gait variability after a cerebellar stroke. Front Neurol. 2016; 7:5–10. DOI: 10.3389/fneur.2016.00084 27313563 PMC4887482

[13] Nascimento LR, de Oliveira CQ, Ada L, Michaelsen SM, Teixeira-Salmela LF. Walking training with cueing of cadence improves walking speed and stride length after stroke more than walking training alone: A systematic review. J Physiother. 2015; 61:10–5. DOI: 10.1016/j.jphys.2014.11.015 25529836

[14] Sohliya L, Thomas R. Rhythmic auditory stimulation for gait training in persons with unilateral transtibial amputation: A randomized-controlled trial. Ann Phys Rehabil Med. 2018; 61:e377. DOI:10.1016/j.rehab.2018.05.875

[15] Hesse S, Konrad M, Uhlenbrock D. Treadmill walking with partial body weight support versus floor walking in hemiparetic subjects. Arch Phys Med Rehabil. 1999; 80:421–7. DOI: 10.1016/S0003-9993(99)90279-4 10206604

[16] Brandt A, Huang HH. Effects of extended stance time on a powered knee prosthesis and gait symmetry on the lateral control of balance during walking in individuals with unilateral amputation. J Neuroeng Rehabil. 2019; 16:1–12. DOI: 10.1186/s12984-019-0625-6 31783759 PMC6883569

[17] Mori H, Tamari M. Predicative factors of the effect of body weight support treadmill training in stroke hemiparesis patients. J Phys Ther Sci. 2020; 32:550–3. DOI: 10.1589/jpts.32.550 32982047 PMC7509156

[18] Crea S, Edin BB, Knaepen K, Meeusen R, Vitiello N. Timediscrete vibrotactile feedback contributes to improved gait symmetry in patients with lower limb amputations: case series. Phys Ther. 2017; 97:198–207. DOI: 10.2522/ptj.20150441 28204796

[19] Sharma A, Torres-Moreno R, Zabjek K, Andrysek J. Toward an artificial sensory feedback system for prosthetic mobility rehabilitation: Examination of sensorimotor responses. J Rehabil Res Dev. 2014; 51:907–18. DOI: 10.1682/JRRD.2013.07.0164 25356723

[20] Sharma A, Leineweber MJ, Andrysek J. Effects of cognitive load and prosthetic liner on volitional response times to vibrotactile feedback. J Rehabil Res Dev. 2016; 53:473–82. DOI: 10.1682/JRRD.2016.04.0060 27532493

[21] Roerdink M, Bank PJM, Peper CLE, Beek PJ. Walking to the beat of different drums: Practical implications for the use of acoustic rhythms in gait rehabilitation. Gait Posture. 2011; 33:690–4. DOI: 10.1016/j.gaitpost.2011.03.001 21454077

[22] Yu L, Zhang Q, Hu C, Huang Q, Ye M, Li D. Effects of different frequencies of rhythmic auditory cueing on the stride length, cadence, and gait speed in healthy young females. J Phys Ther Sci. 2015; 27:485–7. DOI: 10.1589/jpts.27.485 25729197 PMC4339167

[23] Smith JD, Martin PE. Walking patterns change rapidly following asymmetrical lower extremity loading. Hum Movement Sci. 2007; 26:412–25. DOI: 10.1016/j.humov.2006.12.001 17289193

[24] Kodesh E, Kafri M, Dar G, Dickstein R. Walking speed, unilateral leg loading, and step symmetry in young adults. Gait Posture. 2012; 35:66–9. DOI: 10.1016/j.gaitpost.2011.08.008 21903395

[25] Ramakrishnan T, Lahiff C, Reed KB. Comparing gait with multiple physical asymmetries using consolidated metrics. Front Neurorobot. 2018; 12:1–12. DOI: 10.3389/fnbot.2018.00002 29487520 PMC5816825

[26] Van Melick N, Meddeler BM, Hoogeboom TJ, Nijhuis-van der Sanden MWG, Van Cingel REH. How to determine leg dominance: The agreement between self-reported and observed performance in healthy adults. PLoS One. 2017; 12:e0189876. DOI:10.1371/journal.pone.0189876 29287067 PMC5747428

[27] Collins TD, Ghoussayni SN, Ewins DJ, Kent JA. A six degrees-of-freedom marker set for gait analysis: Repeatability and comparison with a modified Helen Hayes set. Gait Posture. 2009; 30:173–80. DOI: 10.1016/j.gaitpost.2009.04.004 19473844

[28] Kadaba M, Ramakrishnan H, Wootten M. Measurement of lower extremity kinematics during level walking. J Orthopeadic Res. 1990; 8:383–92. DOI: 10.1002/jor.1100080310 2324857

[29] Schreiber C, Moissenet F. A multimodal dataset of human gait at different walking speeds established on injury-free adult participants. Sci Data. 2019; 6:111. DOI:10.1038/s41597-019-0124-4 31270327 PMC6610108

[30] Patterson KK, Gage WH, Brooks D, Black SE, McIlroy WE. Evaluation of gait symmetry after stroke: A comparison of current methods and recommendations for standardization. Gait Posture. 2010; 31:241–6. DOI: 10.1016/j.gaitpost.2009.10.014 19932621

[31] Patterson KK, Nadkarni NK, Black SE, McIlroy WE. Gait symmetry and velocity differ in their relationship to age. Gait Posture. 2012; 35:590–4. DOI: 10.1016/j.gaitpost.2011.11.030 22300728 PMC3914537

[32] Armstrong RA. Recommendations for analysis of repeated measures designs: testing and correcting for sphericity and use of MANOVA and mixed model analysis. Ophthalmic and Physiological Optics. 2017; 37:585–93. DOI: 10.1111/opo.12399 28726257

[33] Georgiou T. Rhythmic haptic cueing for gait rehabilitation of hemiparetic stroke and brain injury survivors. Open University. 2018. DOI: 10.21954/ou.ro.0000dabf

[34] Lee S, Lee K, Song C. Gait training with bilateral rhythmic auditory stimulation in stroke patients: A randomized controlled trial. Brain Sci. 2018; 8:164. DOI:10.3390/brainsci8090164 30200282 PMC6162464

[35] Cha Y, Kim Y, Chung Y. Immediate effects of rhythmic auditory stimulation with tempo changes on gait in stroke patients. J Phys Ther Sci. 2014; 26:479–82. DOI: 10.1589/jpts.26.479 24764615 PMC3996403

[36] Roemmich RT, Leech KA, Gonzalez AJ, Bastian AJ. Trading symmetry for energy cost during walking in healthy adults and persons poststroke. Neurorehabil Neural Repair. 2019; 33:602–13. DOI: 10.1177/1545968319855028 31208276 PMC6688943

[37] Robinson JL, Smidt GL. Quantitative gait evaluation in the clinic. Physi Ther. 1981; 61:351–3. DOI: 10.1093/ptj/61.3.351 7465630

[38] Escamilla-Nunez R, Andrysek J. Exploration of vibrotactile biofeedback strategies to modulate spatiotemporal gait asymmetry of individuals with lower-limb amputation. Can Prosthet Orthot J. 2022; 5(1). DOI:10.33137/cpoj.v5i1.36744 PMC10443477 37614481

[39] Thaut MH, Mcintosh GC, Prassas SG, Rice RR. Effect of rhythmic auditory cuing on temporal stride parameters and EMG . patterns in gait of stroke patients. Neurorehabil Neural Repair. 1993; 7(1):9–16. DOI: 10.1177/136140969300700103

[40] Levine D, Richards J, Whittle MW. Whittle's gait analysis. Elsevier health sciences; 2012. Available from: https://www.elsevier.com/books/whittles-gait-analysis/levine/978-0-7020-4265-2

[41] Lin C-C, Whitney SL, Loughlin PJ, Furman JM, Redfern MS, Sienko KH, et al. The effect of age on postural and cognitive task performance while using vibrotactile feedback. J Neurophys. 2015; 113:2127–36. DOI: 10.1152/jn.00083.2014 PMC4416543 25589585

[42] Rossi S, Lisini Baldi T, Aggravi M, Ulivelli M, Cioncoloni D, Niccolini V, et al. Wearable haptic anklets for gait and freezing improvement in Parkinson’s disease: A proof-of-concept study. Neurol Sci. 2020; 41:3643–51. DOI: 10.1007/s10072-020-04485-4 32483689

[43] Cornwell T, Woodward J, Wu M, Jackson B, Souza P, Siegel J, et al. Walking with ears: altered auditory feedback impacts gait step length in older adults. Front Sports Act Living. 2020; 2:1–11. DOI: 10.3389/fspor.2020.00038 PMC7739652 33345030

